# The regulatory role of m6A in cancer metastasis

**DOI:** 10.3389/fcell.2025.1539678

**Published:** 2025-04-28

**Authors:** Ying Zhou, Peng Cao, Qing Zhu

**Affiliations:** ^1^ Division of Abdominal Tumor Multimodality Treatment, Cancer Center, West China Hospital, Sichuan University, Chengdu, China; ^2^ Department of Colorectal Cancer Center, West China Hospital, Sichuan University, Chengdu, China

**Keywords:** M6A, RNA, methylation, metastasis, cancer

## Abstract

Metastasis remains a primary cause of cancer-related mortality, with its intricate mechanisms continuing to be uncovered through advancing research. Among the various regulatory processes involved, RNA modification has emerged as a critical epitranscriptomic mechanism influencing cancer metastasis. N6-methyladenosine (m6A), recognized as one of the most prevalent and functionally significant RNA modifications, plays a central role in the regulation of RNA metabolism. In this review, we explore the multifaceted role of m6A in the different stages of cancer metastasis, including epithelial–mesenchymal transition, invasion, migration, and colonization. In addition to summarizing the current state of our understanding, we offer insights into how m6A modifications modulate key oncogenic pathways, highlighting the implications of recent discoveries for therapeutic interventions. Furthermore, we critically assess the limitations of previous studies and propose areas for future research, including the potential for targeting m6A as a novel approach in anti-metastatic therapies. Our analysis provides a comprehensive understanding of the regulatory landscape of m6A in metastasis, offering directions for continued exploration in this rapidly evolving field.

## Introduction

**FIGURE 1:**
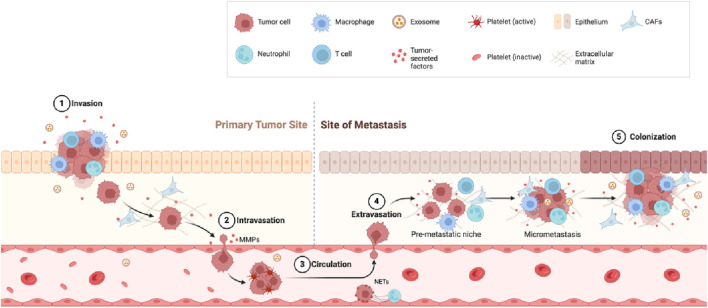
Metastasis steps. Invasion: Tumor cells break through surrounding tissue with help from immune cells such as macrophages and neutrophils. Intravasation: Tumor cells enter the bloodstream by breaking down the tissue barrier using matrix metalloproteinases (MMPs). Circulation: Tumor cells travel through the blood, protected by platelets and other factors. Extravasation: Tumor cells exit the bloodstream and settle in a new site (pre-metastatic niche). Colonization: Tumor cells grow into new tumors at the metastatic site.

A critical phase in cancer progression is the dissemination of malignant cells to distant tissues or organs via the bloodstream or lymphatic vessels, known as metastasis (Karras et al., 2024). This portends a poor prognosis, considering that a minimum of 60% of fatalities associated with cancer can be attributed to the presence of metastasis. Cancer metastasis is a multi-step cellular process, known as the “invasion-metastasis cascade,” which includes a series of processes including local invasion, intravasation, survival in the circulation, arrest at a distant organ site, extravasation, micro-metastasis formation, and metastatic colonization (Valastyan and Weinberg, 2011; Gerstberger et al., 2023) (Figure 1). Research shows that tumor metastasis is regulated by a series of complex networks (Shi et al., 2024), such as epitranscriptome regulation which occurs at the RNA level. The presence of modified nucleotides on RNA molecules, known as RNA modifications, has been known since the 1950s (Cohn, 1960; Littlefield and Dunn, 1958). To date, researchers have discovered more than 170 chemical modifications in various RNA classes in prokaryotes and eukaryotes (Cappannini et al., 2024). These chemical markers exert important influences on various cellular processes in eukaryotes (Chen et al., 2016; Shi et al., 2020). Among them, N6-methyladenosine (m6A) represents the most prevalent and thoroughly investigated modification found in messenger RNA (mRNA) and long noncoding RNA (lncRNA), affecting RNA metabolism and expression by regulating RNA splicing, nuclear export, decay, translation, and stability, and plays a vital role in cancer metastasis (Deng et al., 2023).

Extensive research has been conducted on the tumor invasion-metastasis cascade (Valastyan and Weinberg, 2011; Shi et al., 2024; Steeg, 2016). This article focuses on summarizing the role of m6A in various steps of cancer metastasis and the related mechanisms, while also pointing out the limitations and development prospects of m6A-related research.

### m6A

The m6A modification consists of the insertion of a methyl group at the 6th nitrogen atom of adenine and is the most intensively studied mRNA modification. m6A methylation is present in 20%–40% of all transcripts encoded by mammalian cells (Dubin and Taylor, 1975; Desrosiers et al., 1974). This modification is not only ubiquitous in mammalian mRNA, but also occurs in a variety of non-coding RNAs, including ribosomal (rRNA), transfer (tRNA), small nucleolar (snoRNA), long non-coding (lncRNA), small nuclear (snRNA), circular (circRNA), and microRNA (miRNA) (Sendinc and Shi, 2023). m6A is usually embedded in the sequence 5′-RRACH-3′ and exhibits diverse distribution patterns within eukaryotic transcriptomes. In mammals, m6A modification is preferentially located within the 3′-untranslated region (3′-UTR) adjacent to the stop codon and is also prevalent in long exons, suggesting a potential role in the regulation of mRNA splicing (Schwartz et al., 2014; Dominissini et al., 2012; Meyer et al., 2012). In Arabidopsis mRNA, this m6A modification is found to be enriched in both the 3′- and 5′-UTR (Luo et al., 2014). m6A modification can regulate RNA processing, maturation, splicing, degradation, stability, nuclear export, and translation, thereby regulating RNA expression and function (Sommer et al., 1978; Wang et al., 2015; Xiao et al., 2016; Roundtree et al., 2017; Wang X. et al., 2014).

**FIGURE 2:**
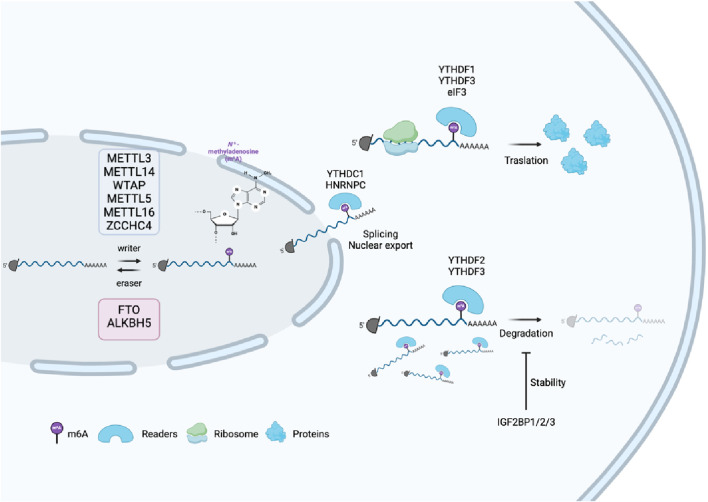
Process of m6A modification and its molecular functions. m6A modification of mRNA is added by “writers” (e.g., METTL3 and METTL14) and removed by “erasers” (FTO and ALKBH5), influencing mRNA fate. m6A-binding “readers,” such as YTHDC1, regulate splicing and export to the nucleus, whereas YTHDF1/3 promote translation in the cytoplasm. Other readers, such as YTHDF2, trigger mRNA degradation, and IGF2BP proteins stabilize mRNA.

The deposition of the vast majority of m6A modifications on RNA depends on the methyltransferase-like protein 3 (METTL3)/METTL14/Wilms’ tumour-associated protein (WTAP) complex; METTL3 is responsible for methylation reactions. METTL14 functions as an allosteric activator; and the guide protein WTAP facilitates the recruitment of the METTL3/METTL14 heterodimer (Liu et al., 2014; Ping et al., 2014). Recently, METTL5, METTL16, and zinc finger CCHC type 4 (ZCCHC4) were found to be novel m6A methyltransferases in eukaryotes (Pinto et al., 2020; van Tran et al., 2019; Su et al., 2022). The demethylation process is controlled by demethylases such as fat mass and obesity-associated protein (FTO) and alkB homolog 5 (ALKHB5) (Jia et al., 2011; Zheng et al., 2013). There is currently no known eraser or reader for METTL5, which is responsible for the m6A modification of 18S rRNA. The elimination of m6A or m6Am by FTO depends on its subcellular distribution. In the nucleus and cytoplasm, the FTO substrate is mainly m6Am and m6A, respectively (Mauer et al., 2017). Different reader proteins can mediate RNA processing, stabilization, translation, and degradation by recognizing m6A modifications. The most famous m6A readers are the YTH domain protein family (YTHDF1, YTHDF2, YTHDF3, YTHDC1 and YTHDC2) and insulin-like growth factor 2 mRNA-binding proteins (IGF2BP) family, the heterogeneous nuclear ribonucleoprotein (HNRNP) protein family, in addition to eukaryotic translation initiation factor 3 (eIF3). YTHDF1, a member of the YTH domain protein family, can interact with m6A modifications located at the mRNA stop codon or 3′-UTR and promote mRNA translation by recruiting eIF3. While there are speculations about the involvement of eIF4G-dependent ring formation in this process, it remains unclear at present how eIF3 is recruited to the stop codon to enhance translation (Wang et al., 2015). Interestingly, a small number of m6A-modified mRNAs are located in the 5′-UTR region, and YTHDF1 directly recruits eIF3 through m6A and assembles the translation initiation complex at the 5′-UTR without the help of cap-binding proteins (Meyer et al., 2015). YTHDF2 mainly regulates the degradation of m6A-modified RNA (Wang X. et al., 2014). YTHDF3 is also thought to promote translation, and some studies have found that it is associated with RNA degradation (Chang et al., 2020; Shi et al., 2017). YTHDC1 promotes pre-mRNA splicing and export through interaction with splicing factors via the m6A modification (Xiao et al., 2016). YTHDC2 is capable of interacting with ribosomal 18S rRNA, which leads to an increase in the translation efficiency of its target genes. Concurrently, this interaction results in a reduction of the abundance of their corresponding mRNA (Hsu et al., 2017; Mao et al., 2019). Proteins of the IGF2BP family contribute to recruiting RNA stabilizers to enhance mRNA stability (Huang et al., 2018). HNRNPA2B1 was shown to selectively recognize methylated pri-microRNAs to promote microRNA maturation (Alarcón et al., 2015a; Alarcón et al., 2015b). HNRNPC is a prevalent nuclear RNA-binding protein that plays a crucial role in the processing of pre-mRNA. m6A modification changes the spatial structure of mRNA and lncRNA to promote the binding of HNRNPC, affecting the alternative splicing of target RNA, thereby affecting gene expression and maturation in the nucleus (Liu et al., 2015) (Figure 2).

### Cancer metastasis

**FIGURE 3:**
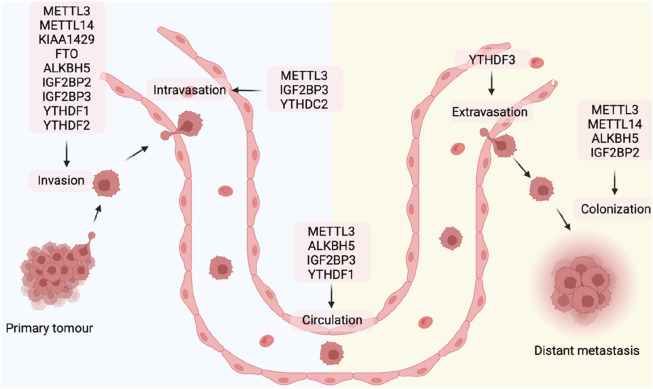
m6A regulators in different steps of tumor metastasis.

Tumor metastasis is formed after a series of complex cellular processes known as the “invasion-metastasis cascade.” Epithelial cells of the original tumor (Karras et al., 2024): locally invade the basement membrane (BM) and extracellular matrix (ECM) (Valastyan and Weinberg, 2011), penetrate the blood vessel (Gerstberger et al., 2023), survive from the vascular transport pressure (Shi et al., 2024), trapped at distant organ locations (Cohn, 1960) undergo extravasation into the parenchymal tissue of distant organs (Littlefield and Dunn, 1958), survive in metastatic tissues to form micro-metastases, and (Cappannini et al., 2024) restart their proliferation program at the metastatic site to give rise to macroscopic, clinically detectable tumors (Valastyan and Weinberg, 2011). The process of tumor metastasis is controlled by the intrinsic molecular signaling pathways of tumor cells, and the interaction between tumor cells and the surrounding environment also plays an essential role. The regulation of m6A modification plays a role in every stage of the process (Figure 3) (Table 1).

**TABLE 1:** Cancer metastasis regulated through m6A methylation in different types of cancer.

Stages of cancer metastasis	Cancer	Model	Type	Regulator	Target	M6A stie and mechanism	Axis	Function	References
Local invasion	Ovarian cancer	Tumor xenograft nude mouse, *in vitro* cell culture	Eraser	ALKBH5	Snail1	CDS/stability	FSH/CREB/ALKBH5/Snail/EMT	Promote metastasis and EMT	Xu et al. (2024)
	Colorectal cancer	Subcutaneous tumors and lung metastases BALB/c nude mouse, *in vitro* cell culture	Writer	METTL3	Snail1	Not mentioned	METTL3/Snail/CXCL2/M2 macrophage	Promote metastasis and EMT	Ouyang et al. (2024)
	Head and neck squamous carcinoma	Popliteal LN metastasis nude mouse, *in vitro* cell culture	Reader	IGF2BP2	Slug/Snail2	CDS/stability	IGF2BP2/Slug/EMT	Promote metastasis and EMT	Yu et al. (2022)
	Breast cancer	*In vitro* cell culture	Eraser	FTO	ZEB1	Not mentioned	piR-17560//FTO/ZEB1	Promotes chemoresistance and EMT	Ou et al. (2022)
	NSCLC	*In vitro* cell culture	Reader	IGF2BP3	TWSIT1	3‘UTR/stability	IGF2BP3/m 6 A/TWIST1	Promote metastasis	Cui et al. (2022)
	HCC	*In vitro* cell culture	Writer	METTL3	lnc-TSPAN12	stability	METTL3/lnc-TSPAN12/EIF3I/SENP1/Wnt/β-catenin	Promote metastasis and EMT	Li et al. (2024)
	Cervical cancer	*In vitro* cell culture	Writer/Reader	METTL3/YTHDF2	CTNNB1	5’UTR/instability	METTL3/YTHDF2/CTNNB1	Promote metastasis	Li et al. (2022)
	Cervical cancer	*In vitro* cell culture	Writer/Reader	METTL3/IGF2BP3	c-MET	Stability	METTL3/IGF2BP3/c-MET/decrease β-catenin membrane localization	Promote metastasis	Li et al. (2022)
	Cervical cancer/Lung cancer	*In vitro* cell culture	Write	METTL3	E2F1	Stability	METTL3/E2F1/decrease CTNNB1 transcription	Promote metastasis	Li et al. (2022)
	Cervical cancer/Lung cancer/Liver cancer	*In vitro* cell culture	Writer/Reader	METTL3/YTHDF1	CTNNB1	5′UTR/inhibit translate	METTL3/YTHDF1/eIF4E1/CTNNB1	Promote metastasis	Li et al. (2022)
	Cervical cancer	Zebrafish *in vivo* study, *in vitro* cell culture	Eraser	ALKBH5	MMP2/9	Increase mRNA and protein level	MALAT1/ALKBH5/MMP2 MMP9	Promote metastasis	Wu et al. (2023)
	HCC	Lung metastasis BALB/c nude mouse, *in vitro* cell culture	Writer	KIAA1429	circDLC1	Decrease RNA level	KIAA1429/circDLC1/HuR/MMP1	Inhibit metastasis	Liu et al. (2021)
	Esophageal squamous cell carcinoma	Subcutaneous xenograft mouse model, *in vitro* cell culture	Eraser	FTO	MMP13	Increase mRNA and protein level	FTO/MMP13	Promote metastasis	Liu et al. (2020)
	Renal cancer	*In vitro* cell culture	Writer	METTL14	P2RX6	Decrease mRNA and protein level	ATP-P2RX6-Ca^2+^ -p-ERK1/2-MMP9	Inhibit metastasis	Gong et al. (2019)
	Melanoma	*In vitro* cell culture	Writer	METTL3	MMP2	Increase protein level	METTL3/MMP2	Promote metastasis	Dahal et al. (2019)
Intravasation	Colorectal cancer	Zebrafish model animals, *in vitro* cell culture	Writer	METTL3	LINC00662/VEGFA	RNA stability	METTL3/LINC00662/VEGFA	Promote angiogenesis	Zhang et al. (2022)
	Gastric cancer	Liver metastasis nude mouse, *in vitro* cell culture	Writer/Reader	METTL3/IGF2BP3	HDGF	mRNA stability	P300/METTL3/IGF2BP3/HDGF	Promote angiogenesis	Wang et al. (2020)
	Bladder Cancer	Mettl3^flox/flox^ and Mettl3 KO mice, *in vitro* cell culture	Writer	METTL3	TEK/VEGFA	Increase protein level	METTL3/TEK/VEGFA	Promote angiogenesis	Wang et al. (2021)
	Colorectal cancer	Subcutaneous xenografts nude mice, *in vitro* cell culture	Reader	IGF2BP3	VEGF	CDS/mRNA stability	IGF2BP3/VEGF	Promote angiogenesis	Yang et al. (2020)
	Lung cancer	Subcutaneous xenografts nude mouse, *in vitro* cell culture	Reader	YTHDC2	VEGFA	5′UTR/translation	YTHDC2/eIF4GI/VEGFA	Promote angiogenesis	Zhang et al. (2023)
Survival in circulation	Malignant glioma	Orthotopic and subcutaneous transplantations C57BL/6 mice, *in vitro* cell culture	Reader	IGF2BP3	CFS3	3′UTRmRNA stability	IGF2BP3/CSF3/NET/NETosis	Promote NET	Dai et al. (2024)
	Melanoma	Lung metastasis Mettl3 knock out C57BL/6 genetic mouse, *in vitro* cell culture	Writer/Reader	METTL3/YTHDF1	SPRED2	CDS/translation	METTL3/YTHDF1/SPRED2/NF-kB STAT3/macrophage polarization	Decrease macrophage polarization and inhibit metastasis	Yin et al. (2021)
Arrest at a distant organ site	Colorectal cancer	Subcutaneous tumors and lung metastases BALB/c nude mouse, *in vitro* cell culture	Writer	METTL3	Snail1	Not mentioned	METTL3/Snail/CXCL2/M2 macrophage	Promote lung metastasis	Ouyang et al. (2024)
	NSCLC	Brain orthotopic transplantation BALB/c nude mice, *in vitro* cell culture	Eraser	ALKBH5	Not mentioned	Not mentioned	BCAT1/ALKBH5/N-cadherin Vimentin/EMT	Promote brain metastasis	Mao et al. (2024)
Extravasation	Breast Cancer	Brain metastasis and orthotopic transplantation nude mice, *in vitro* cell culture	Reader	YTHDF3	ST6GALNAC5/GJA1/EGFR/VEGFA	5′UTR/translation	YTHDF3/ST6GALNAC5/GJA1/EGFR/VEGFA	Promote blood-brain barrier extravasation, angiogenesis, and outgrow	Chang et al. (2020)
Micro-metastasis formation	Breast cancer	Xenograft Mouse Model, *in vitro* cell culture	Reader	IGF2BP2	ZNF281	3′UTR/mRNA stability	lncSNHG5/IGF2BP2/ZNF281/CCL2/CCL5	Promote premetastatic niche formation	Zeng et al. (2022)
Metastatic colonization	Glioblastoma	Foxn1^nu/nu^ athymic nude mice, *in vitro* cell culture	Eraser	ALKBH5	FOXM1	3′UTR	ALKBH5-FOXM1	Promote cancer stem cell growth	Zhang et al. (2017)
	Glioblastoma	Orthotopic intracranial mouse model, *in vitro* cell culture	Writer	METTL3	SOX2	3′UTR/mRNA stability	METTL3/SOX2	Promote cancer stem cell growth	Visvanathan et al. (2018)
	Leukemia	Xenograft NSG Mouse Model, *in vitro* cell culture	Writer	METTL3	MYC/BCL2/PTEN	Translation	METTL3/c-MYC/BCL2/PTEN	Maintenance of the hematopoietic stem cell program	Vu et al. (2017)
	Bladder cancer	*In vitro* cell culture	Writer	METTL14	Notch1	mRNA instability	METTL14/Notch 1	Inhibit tumor initiating cells self-renewal	Gu et al. (2019)

### Local invasion

**FIGURE 4:**
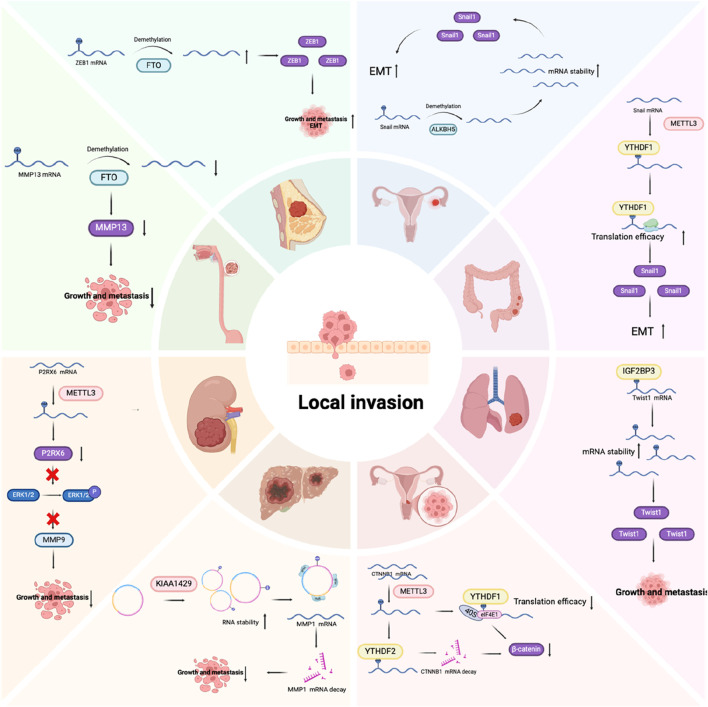
The role of m6A regulators in the invasion stage of tumor metastasis.

Local invasion describes the mechanism by which cancer cells penetrate the BM and invade the adjacent tumor-associated stroma. The writers, erasers, and readers of m6A modification play important roles in this process (Figure 4). The junctions between epithelial cells are an important obstacle to the spread of cancer cells, especially the intercellular junctions mediated by E-cadherin (Berx and van Roy, 2009; Vleminckx et al., 1991). These junctions hold epithelial cells together and prevent detachment of individual epithelial cells from neighboring cells. To surmount this challenge, cancer cells may initiate a key cell biological process called epithelial–mesenchymal transition (EMT). This biological process causes polarized cells to acquire mesenchymal characteristics, with alterations in the expression of cellular adhesion molecules and the cytoskeleton, promoting migration and invasion, increasing tumor stemness, and enhancing resistance to chemotherapy and immunotherapy (Nieto et al., 2016; Yilmaz and Christofori, 2009). The opposite procedure is called mesenchymal–epithelial transition (MET). It is worth noting that both EMT and MET are essential for the metastatic process. EMT enables primary tumor cells to acquire the ability to migrate, whereas MET halts the cellular migration phase, thereby promoting better colonization of tumor cells at distant metastatic sites. In living organisms, EMT plays a role in several physiological and pathological processes such as inflammation, wound repair, tissue fibrosis, cancer progression, and embryonic development (Grande et al., 2015; Gros and Tabin, 2014). Cells exist in a continuum of states with inherent plasticity, progressively acquiring various transformation intermediates, rather than being confined to two polarized cell identities. EMT represents a fluid and progressive transition from the epithelial to the mesenchymal cell phenotype. Tumor cells in the intermediate phases of the EMT process display a combination of epithelial and mesenchymal features, enhancing their ability to survive, spread, and colonize distant metastatic sites. EMT is tightly controlled by a complex regulatory network orchestrated by numerous intrinsic and extrinsic factors, such as various transcription factors (TFs), epigenetic changes, post-translational modifications, and non-coding RNA-mediated mechanisms. These factors drive the mesenchymal shift by downregulating epithelial markers and upregulating mesenchymal-associated markers (Huang et al., 2022; Stemmler et al., 2019).

The major EMT-related TFs (EMT-TFs) are snail family transcriptional repressor 1 (SNAIL1) and SNAIL2/Slug, zinc finger E-box binding homeobox 1/2 (ZEB1/2), and twist family BHLH transcription factor 1/2 (TWIST1/2) (Lamouille et al., 2014). SNAIL1 directly inhibits the formation of E-cadherin and tight junction proteins, while upregulating mesenchymal phenotype markers like fibronectin and vimentin (Dong et al., 2012; Tong et al., 2012). SNAIL2/Slug facilitates the disruption of cell adhesion and polarity, while enhancing migratory and invasive abilities (Casas et al., 2011). ZEB1/2 belong to the human ZEB family. They are zinc finger TFs that attach to regulatory gene sequences at the E-box, either promoting or inhibiting transcription. Notably, ZEB1 and ZEB2 exert contrasting effects on various cell lines, suggesting that their function is highly dependent on the environment. For example, in a melanoma mouse model, ZEB2 inhibits cancer metastasis, whereas ZEB1 promotes tumor initiation and progression (Caramel et al., 2013). In clinical research, reduced ZEB2 levels correlated with lower survival rates in melanoma patients, whereas elevated ZEB1 expression was linked to poorer clinical prognoses (Denecker et al., 2014). TWIST1/2 belong to the basic helix-loop-helix (BHLH) TF family. Like SNAIL, TWIST1 can suppress E-cadherin expression while upregulating N-cadherin, leading to a reduction in cell adhesion and an enhancement in cell motility. This shift plays a crucial role in facilitating cellular migration and promoting metastatic potential in cancer (Yang et al., 2008). m6A modifications have been confirmed to directly affect the expression of these EMT-TFs, thereby affecting the EMT process. For example, Snail1 mRNA itself will be modified by m6A, and its regulation depends on the writing protein METTL3 or the erasing protein ALKBH5. Its final expression is determined by the reading protein YTHDF1 or 2, and thus regulates the expression levels of E- and N-cadherin, promoting the EMT process (Xu et al., 2024; Ouyang et al., 2024). In head and neck squamous cell carcinoma, SNAIL2 is also modified by m6A. IGF2BP2 has the capacity to recognize and attach to m6A-modified sites, and promote its mRNA stability, thereby regulating the EMT process and promoting lymph node metastasis (Yu et al., 2022). Similarly, ZEB1/2 and Twist can also be regulated in an m6A-dependent manner, thereby affecting the EMT process of tumor cells (Ou et al., 2022; Cui et al., 2022; Cai et al., 2024). Research has demonstrated that noncoding RNA are of essence in EMT regulation. lncRNA often acts as a molecular sponge to interact with miRNA, inhibiting the degradation of EMT-TF to regulate EMT (Yuan et al., 2014; Grelet et al., 2017). The m6A modification can indirectly affect EMT by affecting the expression of non-coding RNA. For example, in hepatocellular carcinoma, it was found that the upregulation of METTL3 promoted the deposition of m6A modification on LncRNA Tetraspanin 12 (TSPAN12), enhanced the stability of LncRNA, and upregulated the expression of lncRNA TSPAN12, acting as a scaffold, promoting the interaction of EIF3I and SUMO specific peptidase 1 (SENP1), thus activating the Wnt/β-catenin signaling pathway to promote EMT (Li et al., 2024). Wnt, transforming growth factor-β (TGF-β), PI3K-AKT, Notch, and other signaling pathways are likewise implicated in the EMT regulatory framework. The key molecules in these pathways are also often regulated by m6A (Tan et al., 2022; Liu et al., 2019; Li et al., 2020). In the common Wnt/β-catenin pathway, Wnt protein activates β-catenin to regulate EMT. Catenin beta 1 (CTNNB1) (encoding β-catenin) mRNA itself can be modified by m6A and is associated with METTL3 expression. METTL3 promotes increased YTHDF2-dependent mRNA degradation and YTHDF1-dependent translation inhibition through m6A modification at the 5ʹ-UTR of the CTNNB1 mRNA. METLL3 can also reduce the cell membrane localization of β-catenin through m6A/IGF2BP3/c-MET, thereby reducing E-cadherin. In summary, METTL3 modulates EMT by regulating β-catenin expression and its subcellular localization (Li et al., 2022). It can be seen that the EMT process is regulated by a complex network, and m6A modification plays a critical role in it. Interestingly, we found that the role of m6A modification may be completely different in different tumor types or cells, which shows that the complexity of RNA modification may be determined by different environmental factors, and ultimately the different expressions of target molecules mediated by different readers ultimately determine the direction of EMT.

Loss of the BM means that cancer cells can directly invade the matrix, a process usually achieved by the breakdown of the BM by matrix metalloproteinases (MMPs). In healthy tissues, MMP activity is tightly regulated through transcriptional processes and post-translational modifications. However, tumor cells can subvert this strict control in a variety of ways, ultimately leading to increased MMP function (Kessenbrock et al., 2015; Egeblad and Werb, 2002). In addition to degrading BM and ECM, MMPs can also induce the release of various growth factors in ECM and promote tumor growth (Kessenbrock et al., 2010). Studies have shown that MMPs can also be directly or indirectly regulated by m6A modification, thereby affecting the invasiveness of tumor cells (Wu et al., 2023; Liu et al., 2021; Liu et al., 2020; Gong et al., 2019; Dahal et al., 2019).

Tumor cells penetrate the BM and invade the ECM, creating numerous opportunities for them to access the circulation and disseminate to distant locations.

### Intravasation

**FIGURE 5:**
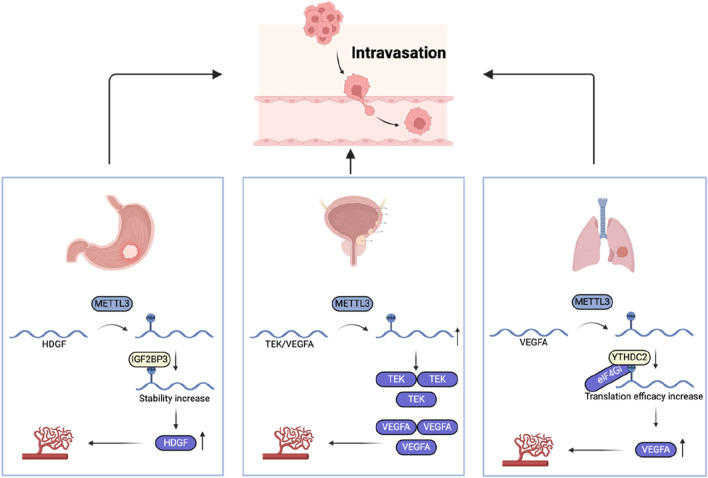
The role of m6A regulators in intravasation.

During metastasis, tumor cells enter the blood circulation through the circulatory or lymphatic system, a process called intravasation. The signaling molecule TGF-β has the ability to amplify the permeability of breast cancer cells (Giampieri et al., 2009). In addition, tumor cell-induced angiogenesis can also promote tumor cells to enter the blood circulation better. Compared with vasculature in healthy tissues, the new blood vessels induced by tumor cells are winding, leaky, and engaged in constant reconstruction, which makes it easier for tumor cells to enter the blood circulation (Carmeliet and Jain, 2011). m6A modification plays a key role in regulating tumor angiogenesis (Figure 5). Researchers have found that METTL3 is upregulated in a variety of tumors, and increased m6A deposition is associated with increased tumor angiogenesis (Zhang et al., 2022; Wang et al., 2020; Wang et al., 2021). In gastric cancer, upregulation of METTL3 is correlated with a poor prognosis for patients. Studies have found that METTL3 upregulates the m6A level of heparin binding growth factor (HDGF) mRNA, and then enhances its stability and promotes its expression through IGF2BP3, thereby increasing tumor angiogenesis and promoting gastric cancer metastasis (Wang et al., 2020). Similarly, in colon cancer, IGF2BP3 has been identified as a regulator of vascular endothelial growth factor (VEGF) mRNA stability via m6A modification, facilitating tumor angiogenesis (Yang et al., 2020). m6A modification in the 5′-UTR region of VEGFA in lung cancer triggers cap-independent translation initiation by recruiting the YTHDC2/eIF4GI complex, increasing its expression and thereby stimulating angiogenesis (Zhang et al., 2023). Once tumor cells successfully enter the blood, they will face more challenges.

### Survival in circulation

Once tumor cells successfully infiltrate the vascular cavity, they can disseminate extensively through the bloodstream. These cells are referred to as circulating tumor cells (CTCs), and they must withstand various pressures to reach distant sites (Headley et al., 2016). Firstly, to detach from the BM, CTCs often undergo a loss of integrin-dependent adhesion, which is usually required for epithelial cell survival. In the absence of attachment to the ECM, epithelial cells often undergo anoikis. Tumor cells can dynamically adapt and resist cell death by constantly sensing and responding to biochemical and biomechanical changes in their microenvironment (Mak et al., 2017; Alanko et al., 2015). Study shows that tumor matrix stiffening can activate mammalian target of rapamycin 1 (mTORC1) and achieve cell death resistance under matrix detachment through the integrin-glycogen synthase kinase 3 beta (GSK3β)-FTO-mTOR axis (Zhang et al., 2024). Secondly, CTCs must also overcome damage caused by the blood flow shear forces and immune cell-mediated attacks. A small number of CTCs have close interactions with platelets, macrophages, neutrophils, cancer-associated fibroblasts (CAFs) or myeloid-derived suppressor cells, which helps them evade the attack of the immune system in the blood and promotes their survival (Rejniak, 2016; Garrido-Navas et al., 2019). Neutrophils are the predominant white blood cells present in the bloodstream and studies suggest they can promote cancer progression (Shaul and Fridlender, 2019). The binding of CTCs to neutrophils is mediated by cell–cell junctions, anchored to the vascular endothelium to resist shear stress extravasation, a process facilitated by a range of cell adhesion molecules, including integrins, cadherins, and surface glycoproteins (Szczerba et al., 2019; Strell et al., 2007; Huh et al., 2010; Rowson-Hodel et al., 2018).

Neutrophil extracellular traps (NETs) are web-like structures composed of histones, DNA, and cytotoxic proteins originating from granules. Tumor cell-induced NETs can promote the survival and metastasis of tumor cells (Adrover et al., 2023). A study found that IGF2BP3 induced NET formation in malignant gliomas and significantly enhanced glioma cell survival (Dai et al., 2024). In liver cancer, METTL5 was found to promote the generation and release of NETs, which may further accelerate the progression of hepatocellular carcinoma. However, the precise mechanism requires further clarification (Wang et al., 2024). Tumor-associated macrophages (TAMs) can also promote the dissemination and extravasation of CTCs (Doak et al., 2018). TAMs actively communicate with CTCs, promoting their survival and aiding in their mesenchymal transformation. They secrete specific cytokines and growth factors, such as TGF-β and interleukin-6, which enhance the EMT process in CTCs. This crosstalk is essential for the mesenchymal CTCs to maintain their invasive potential, allowing them to metastasize to distant organs, such as the liver and lungs (Wei et al., 2019). Moreover, TAMs facilitate the acquisition of enhanced mechanical adhesion properties and increased cellular resilience in CTCs, thereby promoting the formation of protective cell clusters. These clusters provide a survival advantage by conferring resistance to shear stress experienced during circulation in the bloodstream, ultimately supporting their metastatic potential (Osmulski et al., 2021). This study found that TAM can promote lung metastasis of melanoma by downregulating METTL3. Mechanistic studies have demonstrated that the loss of METTL3 results in decreased sprouty related EVH1 domain containing 2 (SPRED2) translation, mediated by YTHDF1. This reduction promotes the activation of nuclear factor kappa-light-chain-enhancer of activated B cells (NF-κB) and signal transducer and activator of transcription 3 (STAT3) signaling pathways, ultimately promoting enhanced tumor growth and metastasis (Yin et al., 2021).

**FIGURE 6:**
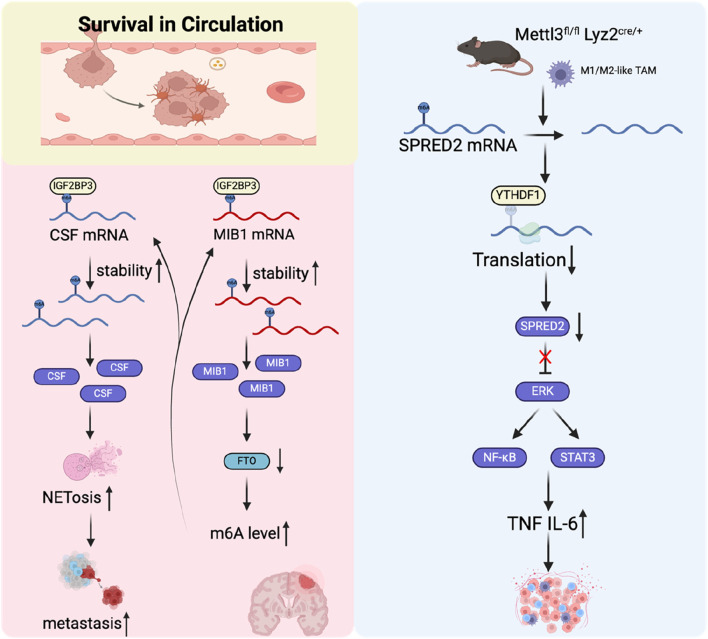
The role of m6A regulators in tumor cell survival in the circulation.

CAFs are one of the most prevalent components of the tumor microenvironment and are crucial in facilitating tumor initiation, promoting angiogenesis, driving metastasis, and contributing to drug resistance (Chen and Song, 2019). In a mouse lung cancer metastasis model, CTCs can carry CAFs from the initial tumor site to the metastatic site (Duda et al., 2010). The study found that m6A and its writer protein METTL3, eraser protein FTO, and reader protein YTHDF2 play an essential role in CAFs promoting tumor cell metastasis and angiogenesis (Liao et al., 2024; Chen et al., 2023). However, its role in CTC survival and immune escape is still unclear. Circulating platelets can bind to CTCs in the bloodstream and form aggregates. CTCs further enhance this aggregate formation by releasing prothrombotic and procoagulant particles or through the expression of tissue factor, which promotes coagulation and may facilitate tumor metastasis (Thomas et al., 2015; Stark et al., 2018). Mediators released by platelets, such as TGF-β, have been shown to induce EMT in CTCs, thereby enhancing their invasive capabilities and promoting metastasis (Labelle et al., 2011). Platelets are thought to protect CTCs from shear stress and induce resistance to apoptosis, facilitating CTC evasion of natural killer (NK) cell attacks through various mechanisms (Egan et al., 2014; Haemmerle et al., 2017; Nieswandt et al., 1999). In general, current studies have revealed that m6A modification is partially involved in CTC circulation survival and immune escape (Figure 6), but further in-depth exploration is still needed.

### Arrest at a distant organ site

**FIGURE 7:**
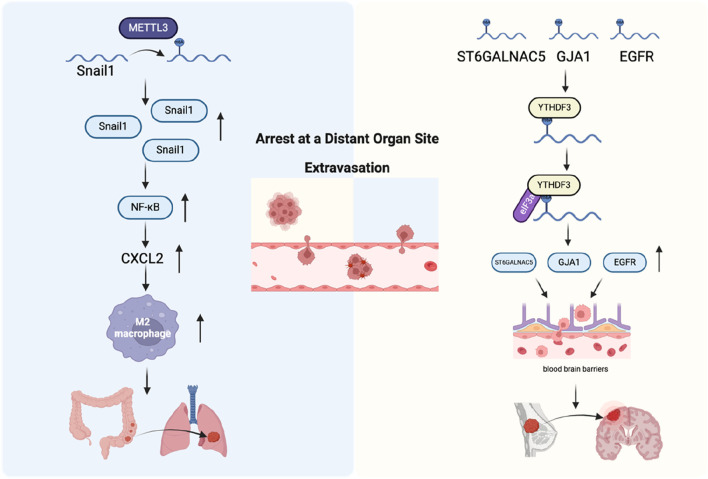
The role of m6A regulators in arrest at a distant organ site and extravasation. Left: Mechanism of colorectal cancer cell metastasis to the lungs. Right: Mechanism of breast cancer cell penetration through the blood-brain barrier and colonization in the brain.

Although tumor cells theoretically have the ability to spread to various organs throughout the body, it has long been noted in clinical practice that different types of tumor cells have different preferences for metastatic target organs (Fidler, 2003). One hypothesis is that the capillary diameter of some organs is not large enough for CTCs to pass through, causing them to stagnate and then metastasize to that organ (Ewing, 1928). Another hypothesis is that CTCs have preferences and can stay in certain tissues by forming specific molecular adhesion interactions with specific tissues (Liu et al., 2017; Fidler and Poste, 2008). In the above process, CTCs as seeds have acquired many metastasis-related abilities, including EMT, tumor stem cells, and tumor-derived or induced cytokines. The microenvironment that is conducive to the survival of CTC metastasis, and its shaping also plays a key role (Liu et al., 2017). Microenvironment remodeling is driven by complex interactions between tumor cells and the ECM, as well as with various cellular components, including TAMs, mesenchymal stem cells, endothelial cells, and tumor-associated fibroblasts. Additionally, the hypoxic conditions within the tumor microenvironment and associated metabolic changes play critical roles in this remodeling process, further supporting tumor progression and metastasis (Joyce and Pollard, 2009). This study revealed that METTL3 and YTHDF1 are highly expressed in tumors from patients with colorectal cancer lung metastasis. METTL3 facilitates the EMT in colorectal cancer by adding m6A modifications to SNAIL mRNA, which enhances its stability. This, in turn, promotes the secretion of CXC motif chemokine ligand 2 (CXCL2) via activation of the NF-κB signaling pathway, contributing to tumor progression and metastasis. Further animal *in vivo* experiments found that secreted CXCL2 promoted the lung metastasis of colon cancer cells by recruiting M2 macrophages (Ouyang et al., 2024). Patients with brain metastases originating from non-small cell lung cancer (NSCLC), accompanied by high levels of branched chain amino acid transaminase 1 (BCAT1) protein were found to have a poor prognosis. Moreover, NSCLC cells with brain metastases had increased branched-chain amino acid (BCAA) catabolism, leading to α-Ketoglutarate (α-KG) depletion. This process resulted in the downregulation of both the expression and activity of the m6A demethylase ALKBH5, thereby impairing its ability to remove m6A modifications from target mRNAs, resulting in increased m6A deposition and elevated expression of mesenchymal-related genes. Consequently, this promoted the EMT in NSCLC cells and facilitated the proliferation of NSCLC cells within the brain, contributing to metastasis and tumor progression (Mao et al., 2024). In summary, RNA modifications play a crucial role in the process of specific tissue transfer, whether in seeds or soil changes (Figure 7).

### Extravasation

During metastasis, tumor cells disseminate to distant organs and tissues through the bloodstream. They then extravasate into the tissue parenchyma by attaching to the endothelial cells of blood vessels and passing through the vessel walls to the metastatic site. This process is called extravasation (Reymond et al., 2013). Vascular extravasation and intravasation are not just opposite but also two mechanistically distinct states. The same types of cells may exist in the tumor microenvironment at the primary tumor site and the secondary metastatic site, but their functions may be completely different (Qian and Pollard, 2010). The permeability of newly formed blood vessels in the primary tumor site is very strong, which provides favorable conditions for tumor cell infiltration. However, in normal tissues at distant metastatic sites, vascular tissues are normalized, which greatly hinders the extravasation of tumor cells. Therefore, tumor cells destroy the normal connection of vascular endothelium by secreting protein angiopoietin-like 4 (Angptl4) and various cytokines, such as cyclooxygenase-2 (COX-2), epiregulin (EREG), and MMP-1/2, thereby promoting the extravasation of tumor cells (Padua et al., 2008; Gupta et al., 2007a). The study found that YTHDF3 expression increased in patients with brain metastases from breast cancer, whereas its depletion impaired the formation of brain metastases and prolonged the survival of mice. YTHDF3 can promote breast cancer cell adhesion and extravasation across the brain endothelium by enhancing interactions between cancer cells and astrocytes, as well as promoting angiogenesis in the context of breast cancer brain metastasis. This process contributes to the establishment and progression of metastatic tumors in the brain. Further studies found that YTHDF3 promoted the translation of epidermal growth factor receptor (EGFR), gap junction protein alpha 1 (GJA1), and ST6 N-acetylgalactosaminide alpha-2,6-sialyltransferase 5 (ST6GALNAC5) in an m6A-dependent manner, which ultimately led to an increase in breast cancer brain metastasis (Chang et al., 2020) (Figure 7).

### Micrometastasis formation

**FIGURE 8:**
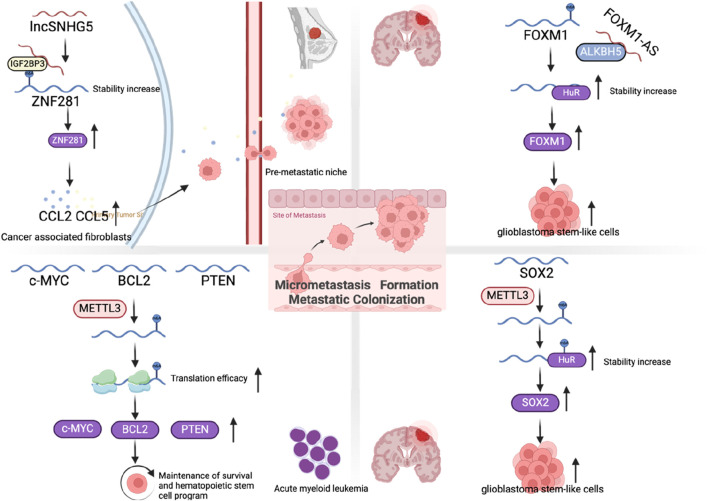
The role of m6A regulators in micrometastasis formation and metastatic colonization.

After tumor cells pass through blood vessels, to form micrometastasis, they need to survive in the unfamiliar microenvironment of the distant metastatic site. The microenvironment at metastatic sites typically exhibits significant distinctions compared to that of the primary tumor. These differences encompass variations in stromal cell populations, ECM composition, the presence of growth factors and cytokines, and even the tissue structural organization. Tumor cells can use complex mechanisms to change the pre-metastatic niches of distant metastases before metastasis occurs, so that they can survive and form small micrometastasis after metastasis is established (Peinado et al., 2017). Tumor cells can affect the distant metastatic microenvironment in advance by secreting cytokines and exosomes. Pre-metastatic niches formation first changes the vascular permeability, followed by changes in fibroblasts in the ECM, or recruiting bone marrow-derived cells to distant metastatic sites, remodeling the ECM, and ultimately attracting CTCs to reside (Joyce and Pollard, 2009; Psaila and Lyden, 2009; Kaplan et al., 2005). In breast cancer, long noncoding RNA small nucleolar host RNA 5 (lncSNHG5) is markedly overexpressed in CAFs. It stabilizes zinc finger protein 281 (ZNF281) mRNA by interacting with the m6A-binding protein IGF2BP2, resulting in the upregulation of CC motif chemokine ligand 2 (CCL2) and CCL5 through ZNF281. This activation triggers the p38 mitogen-activated protein kinases (P38 MAPK) pathway in endothelial cells, facilitating neovascularization and increasing vascular leakage, thereby contributing to the establishment of the pre-metastatic niche (Zeng et al., 2022) (Figure 8).

### Metastatic colonization

If disseminated tumor cells successfully survive from their first encounter with the unfamiliar microenvironment of the metastatic site, this does not necessarily mean that they are guaranteed to proliferate and form macroscopic metastases. The overwhelming majority of disseminated cells are either slowly exhausted over a period of weeks to months or persist in the form of microcolonies in an apparently long-term dormant state (Gomis and Gawrzak, 2017; Sosa et al., 2014). Another key factor for successful metastatic colonization is the self-renewal ability of tumor cells. Studies suggest that there is a group of cells with high self-renewal ability inside tumor cells, called tumor-initiating (TICs) or tumor stem cells (Shackleton et al., 2009; Clevers, 2011). Previous studies have found that EMT can promote the transformation of tumor cells into TICs (Ye et al., 2015). Furthermore, the TFs from the inhibitor of differentiation (ID) family, such as ID1 and ID3, along with the homeobox TF NK2 homeobox 1 (NKX2-1), are capable of modulating the TIC phenotype in cancer cells (Ullmann et al., 2018; Winslow et al., 2011; Gupta et al., 2007b). A study revealed that m6A modification plays a critical role in regulating embryonic stem cells to maintain their self-renewal capacity (Figure 8). In mouse embryonic stem cells, the knockdown of METTL3 and METTL14 results in a loss of this self-renewal capability, highlighting the importance of these m6A-related enzymes in stem cell maintenance (Wang Y. et al., 2014). Interestingly, another study suggested that mouse embryonic stem cells that lose the m6A modification cannot completely terminate their primitive pluripotency and their differentiation potential is also limited, resulting in early embryonic death (Geula et al., 2015). In cancer stem cells, studies have shown that the depletion of FTO and ALKBH5 leads to increased m6A modification, which can significantly reduce the self-renewal ability of cancer stem cells (Zhang et al., 2017; Su et al., 2020). However, in glioma stem-like cells, it was found that METTL3 expression increased, promoting sex determining region Y-box 2 (SOX2) mRNA m6A modification immersion and increased stability, thereby maintaining its stem cell characteristics (Visvanathan et al., 2018). Similarly, in acute myeloid leukemia, depletion of METTL3 promotes cell differentiation while reducing cell proliferation (Vu et al., 2017). In contrast, in bladder cancer, it was reported that METTL14 can inhibit bladder TIC’s self-renewal ability and tumorigenesis through m6A modification of Notch1 (Gu et al., 2019). Overall, these results suggest that changes in m6A levels can significantly affect cell fate and differentiation state, but these effects may depend on the cellular context as well as the differentiation state of the cells themselves.

## Conclusion and future outlook

In this review article, we mainly focus on the regulatory role of m6A modification in various stages of tumor metastasis. Tumor metastasis is a complex and inefficient process. Abnormal RNA m6A modification is closely related to tumor metastasis, which provides us with additional means and methods to interfere with tumor metastasis. In different processes of metastasis or types of tumors, m6A modification may have completely different effects. This shows that the type of effect that m6A modification exhibits is environment- and time-dependent. Writing proteins and erasing proteins are regulated by different mechanisms, and the ultimate role of m6A modification is also determined by different reading proteins. While existing studies have made significant progress in elucidating the cellular and molecular processes that propel tumor metastasis and new insights and mechanisms continue to emerge, our understanding of this process is still incomplete. The underlying mechanism of metastasis coordination by this modification remains to be further clarified. The discovery of these mechanisms provides significant potential for clinical application.
